# Circumflex Coronary Artery Injury during Modern Mitral Valve Surgery—A Review of Current Concepts and Perspectives

**DOI:** 10.3390/medicina59081470

**Published:** 2023-08-16

**Authors:** Johan van der Merwe, Filip Casselman

**Affiliations:** 1 The Keyhole Heart Centre, Netcare Blaauwberg Hospital, Cape Town 7441, South Africa; drjohan@acvti.co.za; 2 Cardiovascular Surgery, Cardiovascular Centre, OLV Clinic, 9300 Aalst, Belgium

**Keywords:** mitral valve surgery, coronary artery injury

## Abstract

The devastating impact of a circumflex coronary artery (CX) injury during mitral valve (MV) surgery is well reported. Despite significant improvements in preoperative risk assessment, intraoperative diagnosis and perioperative treatment strategies of CX injury during MV surgery, recent reports re-emphasize the variability in presentation, the unpredictable mechanisms of injury and the conflicting evidence regarding perioperative management. The progressive transition from conventional sternotomy access to minimally invasive surgical and transcatheter (TC) interventions for MV disease are associated with significant learning curves and require additional single-shaft and robotic console suture manipulation skills with special attentiveness to the potential risk of CX injury. The introduction of hybrid theatres that facilitate single stage surgical and TC interventions also provides new intraoperative diagnostic and therapeutic options without transporting unstable patients for percutaneous coronary intervention (PCI) assessment. By utilizing a MeSH terms-based PubMed search, a total of 89 patients with CX injury that occurred during MV surgery was identified from 49 reports between 1967 and 2022. MV surgery was performed by conventional sternotomy (*n* = 76, 85.4%), endoscopic (*n* = 12, 13.4%) and robotic access (*n* = 1, 1.1%), with 35 injuries (39.3%) resulting in total CX occlusion. Rescue PCI was utilized in 40 patients (44.9%). This manuscript provides a systematic overview of all available historic and contemporary reports on CX injury during MV surgery, outlines recent refinements in CX injury mechanisms, describes current MV surgery associated CX injury prevention and diagnosis and treatment strategies and highlights important MV procedural aspects that may minimize the risk and consequences of CX injury.

## 1. Introduction

**Figure 1:**
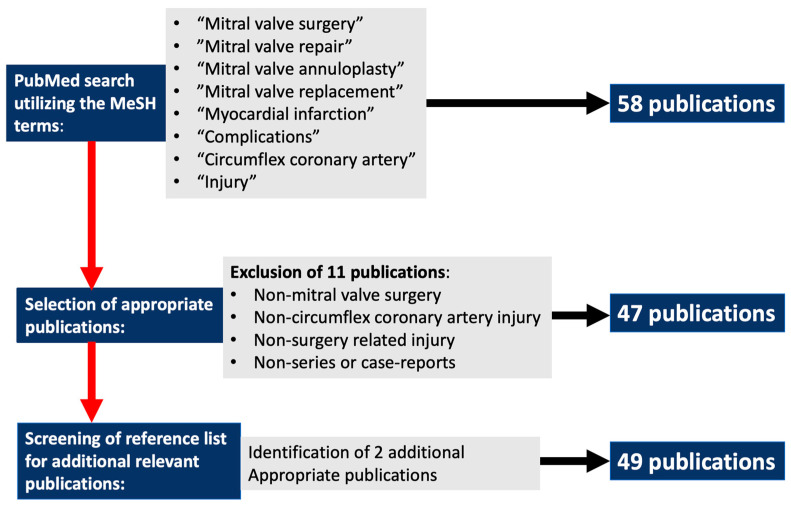
Flowchart outlining the PubMed search methodology.

Myocardial infarction secondary to circumflex coronary artery (CX) distortion or occlusion during mitral valve (MV) surgery is associated with devastating postoperative morbidity and mortality outcomes [1,2,3,4,5,6,7,8,9,10,11,12,13,14,15,16,17,18,19,20,21,22,23,24,25,26,27,28,29,30,31,32,33,34,35,36,37,38,39,40,41,42,43,44,45,46,47,48,49]. Despite significant improvements in preoperative risk identification [50,51,52,53,54,55,56,57,58,59,60], surgical techniques [61,62,63,64,65] and surgical awareness, CX injury during MV surgery remains a dreaded complication with no current consensus on preventative and therapeutic strategies. This manuscript provides a systematic review of all available reports on CX injury during isolated MV surgery and describes current CX injury prevention and diagnosis and treatment strategies. Less invasive MV surgical approaches that utilize special single shafted instruments under direct, endoscopic or robotic vision [66] require additional instrument manipulation skills [67,68,69] and a thorough understanding of the CX, coronary sinus (CS) and MV surgical anatomy to avoid CX injury during the initial learning curves. Furthermore, reports on the potential incidence and risk of CX injury related to advances in innovative transcatheter MV repair [70,71] and replacement [72,73] technology are also emerging. By utilizing the MeSH terms “mitral valve surgery”, “mitral valve repair”, “mitral valve annuloplasty”, “mitral valve replacement”, “circumflex coronary artery injury”, “complications”, “myocardial infarction” in various combinations, a total of 89 patients who sustained CX injury secondary to MV surgery was identified from 49 PubMed reports between 1967 and 2022 (Table 1). Between 2011 and 2022, a total of 29 reports described 60 CX injury events during MV surgery. A flowchart that outlines the PubMed and PRISMA search methodology is presented in Figure 1.

## 2. Current Circumflex Coronary Artery Injury Prevention, Diagnosis and Treatment Strategies

The documented incidence of CX injury secondary to MV surgery varies between 0.15% and 4.0% [1,2,3,4,5,6,7,8,9,10,11,12,13,14,15,16,17,18,19,20,21,22,23,24,25,26,27,28,29,30,31,32,33,34,35,36,37,38,39,40,41,42,43,44,45,46,47,48,49], but various authors suggest that the true incidence is probably significantly higher due to underreporting. Danielson and colleagues [1] were the first to document 3 patients with CX injury in their series of 178 MV procedures in 1967, followed by Roberts [2], Morin [3], Virmani [4] and various other authors describing their experiences. The recent advances in minimally invasive and TC mitral valve technology and techniques are paralleled by renewed interest and awareness of CX injury during MV procedures, with contemporary reports outlining innovative strategies to prevent, timeously diagnose and optimally treat CX injury [31,48].

A. Current strategies to identify circumflex coronary arteries at risk during mitral valve surgery

**Figure 2:**
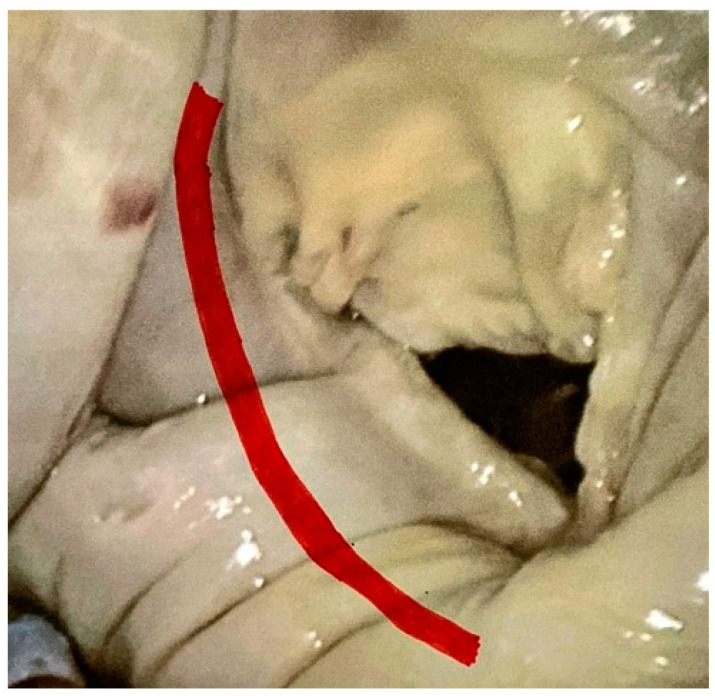
Expected circumflex artery course in relation to the posterior mitral valve annulus during endoscopic mitral valve repair.

Bennani and colleagues [60] recently described the surgical anatomy of the CX–CS–MV complex after dissecting and measuring the course and distances of the CX, CS and MV in 25 explanted hearts. The CX usually courses between the base of the left atrial appendage and the anterior MV commissure, 3–4 mm from the MV leaflet–annular attachment and progressively courses further away from the posterior MV annulus (Figure 2). Table 2 summarizes clinical and contemporary imaging criteria considered to be high risk for CX injury within the context of MV procedures.

**Figure 3:**
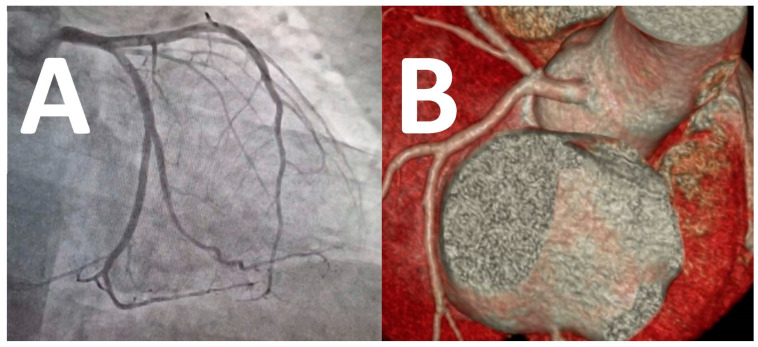
A coronary angiogram (**A**) and computerized tomographic coronary angiographic 3D reconstruction (**B**) of a large left dominant circumflex system at risk during mitral valve surgery.

- Coronary angiography;

Preoperative coronary angiography is regarded as the gold standard for coronary artery evaluation within the context of valvular heart disease [74,75]. The identification of anomalous [2,5,76], large left dominant (Figure 3A) or codominant CX systems predispose to a higher risk for CX injury [48,59].

- Computerized tomography coronary angiography;

Numerous reports describe the expanding role of computerized tomographic coronary angiography (CTA) in the evaluation of the CX–CS–MV complex. Mlynarski and colleagues [58] reported 52 anatomical variations in 320 CTA evaluations of the CX–CS–MV complex and reported that only 1.6% of CX could not be visualized in their series. The authors concluded that the significant anatomical variation of the CX strengthens the role of CTA in preoperative surgical and transcatheter MV planning. Caruso and colleagues [59] recently concluded from their series of 95 examinations that the addition of 3D reconstruction to preoperative CTA facilitates improved and operator-independent distance measurement accuracy between the CX and MV annulus (Figure 3B) and regard CTA with 3D reconstruction superior to other imaging modalities in identifying CX potentially at risk during MV surgery. The measured distance between the CX and MV annulus was significantly smaller in left (mean distances of 3.0 ± 2.1 mm) and codominant CX systems (4.6 ± 2.3 mm) compared to right dominant CX systems (mean distances of 5.9 ± 3.2 mm). However, the authors also describe that more than 20% of right dominant CX systems presented with CX to MV annular distances of less than 3 mm, which concur with earlier reports from Virmani [4], Cornu [76] and Kaklikkaya [77]. Only reports from Mulpur [7], Grande [18] and Varela [21] describe CX injury during MV surgery in a dominant right coronary artery system. CTA is now well established in the diagnosis of coronary artery disease [55], and its role in evaluating coronary artery anatomy and disease as a primary imaging modality in valvular heart disease will continue to expand.

- Transthoracic and transesophageal echocardiography;

Transthoracic echocardiography (TTE) can identify posterior MV leaflet pathology that requires extensive reconstructions in close relation to the CX anatomical course. Severe MV annular calcification and destructive posterior MV or annular infective endocarditis are amongst the TTE-identifiable diseases that present increased CX injury risk during MV surgery. The CX course in relation to the MV can also be appreciated by TTE. Krzanowski and colleagues [56] described their TTE technique to visualize the proximal and middle CX course by parasternal short axis and modified five-chamber TTE views. The authors suggest that CX evaluation by TTE can be of value in evaluating the CX–CS–MV complex, but acknowledge that advanced TTE skills are required and conclude that additional, non-operator-dependent imaging modalities should be used to guide CX anatomy in preparation for MV surgery. Bevilacque [51] and Mak [53] independently reported on the excellent CX and CX–CS–MV complex visualization by transesophageal echocardiography (TEE) and 3D imaging software, which facilitates accurate distance measurement capabilities that correlate well with current CTA technology. Ender and colleagues [57] routinely visualize the proximal and distal CX course with its associated diameter in relation to the CX–MV complex during MV surgery by using a combination of B-mode imaging and color Doppler. The authors reported successful proximal, CX coronary sinus intersection and distal CX visualization in 99%, 90% and 86% of patients, respectively. A modified mid-esophageal long-axis view of the aortic valve at a 110 ± 20 degrees transducer angle is regarded as the optimal TEE view for accurate CX diameter measurements along its course. The important role of TEE skilled anesthetists in ensuring optimal MV surgery outcomes were recently re-emphasized by Landa and colleagues [46]. Man and colleagues [54] described the application of CTA and TEE merger software to further refine the CX–CS–MV complex relationships in cases where CTA-derived measurements identify anatomy at risk. Experts currently suggest that the combination of TTE, TEE and CTA provides exciting multi-modality imaging to determine CX injury risk in preparation for less invasive and TC mitral valve interventional planning [48,54,57,59]. Further studies are required to define the role of each individual imaging modality in isolation and in combination to evaluate CX anatomy and to redefine the traditional role of routine coronary angiography at the expense of additional radiation exposure in preparation for contemporary MV surgery.

B. Modern mitral valve surgical techniques and technology to minimize circumflex coronary artery injury risk

**Figure 4:**
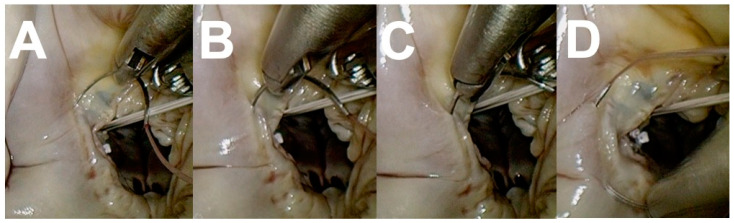
The principles of posterior mitral valve annuloplasty suture placement include (**A**) a 90 degree annular angle entrance (**B**) in parallel with the mitral valve annulus. (**C**) Careful driving of the needle through the appropriate depth and (**D**) careful needle extraction along its curve.

The rapid development, favorable outcomes and simplification of durable MV repair techniques as described by Carpentier [62,63,64] redefined the modern role of MV replacement [61]. The reported decrease in MV replacement procedures performed in developed countries [78] parallels modern international guidelines [74,75] that strongly advocate MV repair whenever possible. Various reports describe CX injury during MV repair [6,8,9,10,11,12,13,14,15,16,17,18,19,20,21,22,23,26,27,29,30,31,32,33,34,35,38,39,41,42,44,46,48,49] and emphasize the importance of accurate and meticulous annuloplasty needle entry angle, direction, depth, instrument manipulation and exteriorization in areas where the CX–CS–MV complex is at risk. Wide posterior leaflet quadrangular resection, extensive posterior sliding plasty and the use of excessively small or large annuloplasty rings should be avoided to minimize the risk of tissue traction, distortion or external compression of the CX in high-risk areas [79,80,81,82,83]. Caruso and colleagues [59] utilized flexible annuloplasty rings in high-risk CX–CS–MV-complex patients, which accounted for 50% of their series. Annular sutures between the anterolateral commissure and P1 were omitted in 58% of these patients. Chauvette and colleagues [83] strongly advocate anterolateral trigone stabilization to prevent partial annuloplasty as a fundamental principle of safe and durable MV repair, emphasized that the fear of CX injury should not result in inadequate MV repair and reiterated the importance of rigid rings within the context of ischemic MV disease. In addition to mechanical CX injury, Obarski and colleagues [84] suggested that water-testing of valve competence following MV repair may potentially result in air embolism and transient CX ischemia, which may resolve or evolve to infarction. Minimally invasive and robotic approaches are becoming increasingly established as excellent surgical alternatives to conventional MV surgery by sternotomy access [66]. However, the learning curves associated with establishing less invasive surgical programs are well reported [67,68,69] and require additional skills in single-shaft and robotic console instrument manipulation. CX injury during less invasive MV procedures is described [13,18,20,26,28,31,41,42,44,46]. The technical aspects of endoscopic posterior annular suture placement are outlined in Figure 4 and can be mastered in innovative simulation environments [85]. Everting and non-everting annular sutures should not be placed more than 3 mm from the posterior annulus in MV replacement. Extensive posterior annular resections and reconstructions required in severe MV annular calcification [86] and destructive infective endocarditis [87] should be performed with CX injury awareness. Innovative TC–MV technology currently includes various annuloplasty devices that use anchors or sutures for annular implantation through the left atrium or CS [70,71] and replacement technology [72,73] that are implanted through peripheral or trans-apical access. A meta-analysis by Kargoli and colleagues [72] recently reported that no CX injury secondary to TC mitral valve devices was described or observed to be up to date. Effectiveness and durability are the main concerns of TC approaches, and outcome reports are progressively emerging with exciting prospects for future treatment of MV disease.

C. Intraoperative circumflex coronary artery injury diagnosis and treatment pathways

Pessa and colleagues [50] emphasized that the clinical presentation of CX injury correlates with the impact on CX flow and the underlying myocardial reserve. The inability to wean from cardiopulmonary bypass, acute hemodynamic compromise, ST-segment elevation on electrocardiography, refractory ventricular arrhythmias, lateral left ventricular regional wall motion abnormalities and disproportionate cardiac enzyme leak can present during or after the separation from cardiopulmonary bypass or later in the postoperative period [31,48]. The reported physiological classification and anatomical mechanisms that result in iatrogenic CX injury are outlined in Table 3.

**Figure 5:**
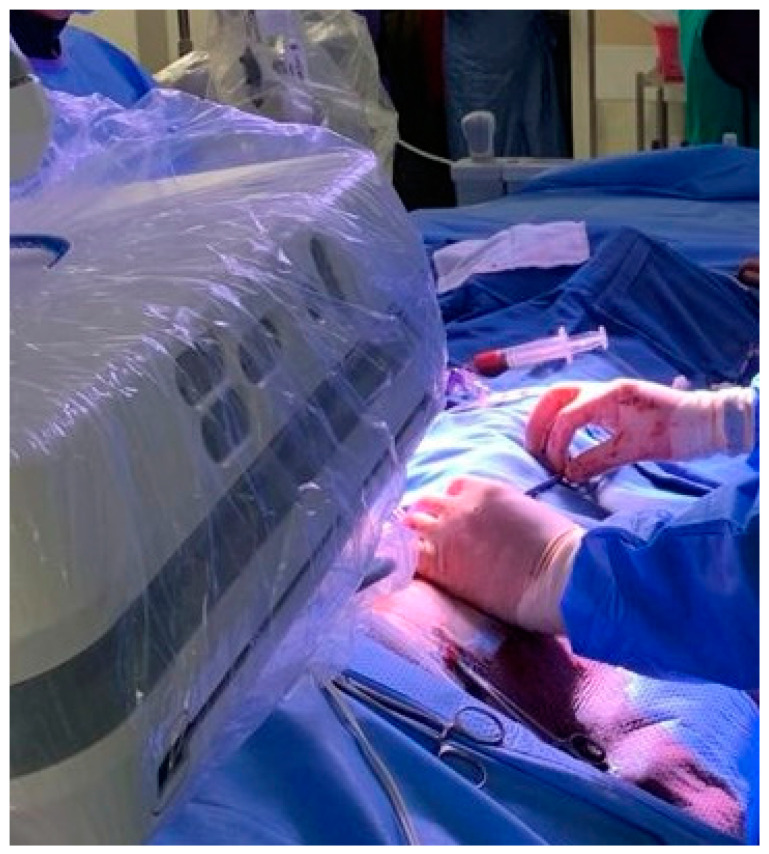
Simultaneous minimally invasive valve surgery and percutaneous coronary interventions in a modern hybrid cardiovascular theatre.

The intraoperative diagnosis of CX injury is currently documented in 20 reports of 34 patients [6,10,11,13,14,16,17,18,19,20,22,28,31,35,39,40,41,42,45,46] and suggests that only 48.3% of all documented CX injuries during MV surgery are diagnosed intraoperatively. Once suspected, urgent confirmation of CX injury and the rapid identification of the responsible mechanism by TEE and/or coronary angiography are crucial to expedite the appropriate treatment strategy [10,11,12,13,14,15,16,17,18,19,20,21,22,23,24,25,26,27,28,29,30,31,32,33,34,35,36,37,38,39,40,41,42,43,44,45,46,47,48,49]. Flow-limiting CX injuries diagnosed intraoperatively require emergency revascularization and restoration of distal perfusion by either emergency empiric coronary artery bypass grafting (CABG) of the obtuse marginal branches utilizing saphenous vein [6,10,14,15,19,27,31,37,38,48], by revising the prosthesis sutures [6,7,17,20,22,30,32,36,38,42,48] or by PCI [8,9,11,12,13,16,18,20,21,22,23,24,26,28,29,31,32,33,34,35,37,38,39,40,41,42,43,44,45,46,47,48,49]. CABG should strongly be considered in an unstable intraoperative setting, where transfer to the catheterization laboratory is prohibited or delayed and where TEE or angiography confirm total CX occlusion [48], especially if sternotomy access is utilized. Revision of annuloplasty sutures in isolation or in combination with either CABG or PCI is documented in 14 reports and can be considered in minimally invasive MV surgery where conversion to sternotomy is not possible or desirable, or where emergency CABG may be challenging [13,18,20,26,28,31,41,44]. Various authors elected to transfer confirmed CX injuries directly from the operating room (*n* = 21) or from a postoperative intensive care setting (*n* = 18) for coronary angiography and PCI after Mantilla and colleagues [8] reported the first PCI for a partially occluded CX injury in 2004. Revascularization by PCI is feasible and preferred in a stable intraoperative setting where CX injury is confirmed to be the result of a partial CX occlusion and where transfer and ischemic reperfusion times are minimal. The inability to cross totally occluded CX injuries with guidewires, stent under-expansion and potential CX rupture are amongst the immediate risks reported with PCI [43,48,49]. The recent advances in hybrid theatre technology, which facilitate combined single-stage surgical MV and TC interventions [65], may decrease CX ischemic reperfusion time by providing the option of rapid on-table coronary angiography and attempts at PCI as an alternative to CABG once the clinical suspicion and mechanism of CX injury is confirmed by TEE (Figure 5).

D. Postoperative and delayed circumflex coronary artery injury diagnosis and treatment strategies

Available reports suggest that the majority of CX injuries secondary to MV surgery manifest during the postoperative period, with 47 of 89 currently documented patients (52.8%) reported to be diagnosed and treated postoperatively. The postoperative clinical presentation of CX injury includes acute or progressive hemodynamic compromise, increased exogenous inotropic support requirement, ST segment elevation or new refractory ventricular arrhythmias on electrocardiography, new lateral left ventricular regional wall motion abnormalities and cardiac enzyme levels suggestive of myocardial ischemia [31,48]. Zegdi and colleagues [15] reported the only postoperative diagnosis of CX injury treated by emergency CABG in 2007, while revision of MV sutures was utilized in four patients with CX injury diagnosed in the postoperative period (8.5%). PCI was the preferred strategy in 46.8% (*n* = 22) of postoperative CX injury patients. Grande [18] and Pettinari [29] independently emphasized the technical difficulty of postoperative PCI in anomalous vessels and advocate careful preoperative MV planning in the event of postoperative CX injury. Reports of delayed complications following rescue PCI are emerging and include in-stent restenosis and a need for future repeat revascularization reinterventions [26,48]. Somekh and colleagues [26] described an iatrogenic CX to left atrial fistula following a PCI for CX injury secondary to MV surgery on the 15th postoperative day. The patient presented with progressive cardiac failure, partial annuloplasty ring dehiscence, severe left atrial enlargement and a fistula. The patient underwent subsequent MV replacement and suture closure of the fistula with a favorable short-term outcome. Gentry and colleagues [36] reported a CX to left atrial fistula 1 year postoperatively, with successful subsequent redo-MV replacement and a favorable outcome. Ziadi and colleagues [27] described the presentation of a large left ventricle pseudoaneurysm and severe MV regurgitation recurrence 5 months after initial MV repair and unrecognized CX injury. The authors postulate that the occlusion may have developed gradually, suggesting that this complication resulted from a combination of fixed mechanical suture occlusion, sub-intimal hematoma, formation, CX spasm and deformation of the vessel. The surgical repair included patch closure of the pseudoaneurysm, mitral valve replacement and tricuspid annuloplasty with a favorable prognostic outcome. Mulpur and colleagues [7] presented a CX injury identified by coronary angiography 14 years after the initial MV surgery. The authors postulate that a suture laceration resulted in an external hematoma around the CX origin in a right dominant coronary artery system and resulted in partial occlusion.

E. The impact of CX injury on contemporary in-hospital outcomes

Only seven reports on CX injury during MV surgery were present up to the year 2000 [1,2,3,4,5,6,7], which included nine MV replacements and two annuloplasty repairs. None of these patients survived the incident prior to 1982, with Tavilla and colleagues [6] being the first to recognize CX injury after annuloplasty intraoperatively and managing the event successfully by combining annuloplasty revision and CABG. Speziale [5] reported a successful 30-day survival without any intervention, but acknowledge poor quality life resulting from irreversible cardiac failure. The progressive awareness of CX injury related to MV surgery, paralleled by improved diagnostic imaging and therapeutic options, resulted in 42 subsequent groups sharing their cumulative experiences that include 89 patients up to date. In total, 30-day mortality occurred in 20.2% of documented patients who sustained CX injury during MV surgery from 1967, which improved to 16.7% from reports published between 2011 and 2022. The favorable in-hospital survival outcomes of conservative therapy in partial CX occlusions were recently independently reported by Yavari [43] and Nasseradine [49] after attempts at rescue PCI were unsuccessful. However, post-discharge reports on these patients were not available. Raza [13], Grande [18], Ender [20], Folkmann [28], Felekos [41], Caruso [42] and Landa [46] described CX injury after endoscopic MV surgery without any 30-day mortality after successful PCI or revision of the annuloplasty. Aravelo [44] described CX injury after robotic MV surgery, which was successfully managed by PCI. Bargagna and colleagues [48] reported 10 patients with CX injury in their series of 6501 MV procedures over a 13-year period. The diagnosis was confirmed intraoperatively and postoperatively in five patients respectively. PCI was performed in distorted or partially occluded CX injuries (*n* = 5) and in one patient with a totally occluded CX. Severe PCI complications occurred in three patients due to coronary artery rupture (*n* = 2) and balloon under-expansion (*n* = 1), which required emergency CABG in two patients. In contrast to PCI, no complications were observed in patients who were treated by immediate CABG (*n* = 3). One patient with CX distortion was treated by removing sutures from the anterolateral commissure to the middle of the posterior annulus with immediate restoration of flow. The authors demonstrated prolonged intensive care admission, blood transfusion requirements and risk of multi-organ dysfunction in all patients. Eight patients were eventually discharged to cardiac rehabilitation centers and two patients died of multi-organ failure and massive cerebral hemorrhage while awaiting heart transplantation, respectively. Severe MV regurgitation was diagnosed 1 year postoperatively and required MV replacement with subsequent mortality due to multi-organ failure.

F. Proposal of a comprehensive algorithm to prevent, diagnose and treat circumflex artery injury during mitral valve surgery

**Figure 6:**
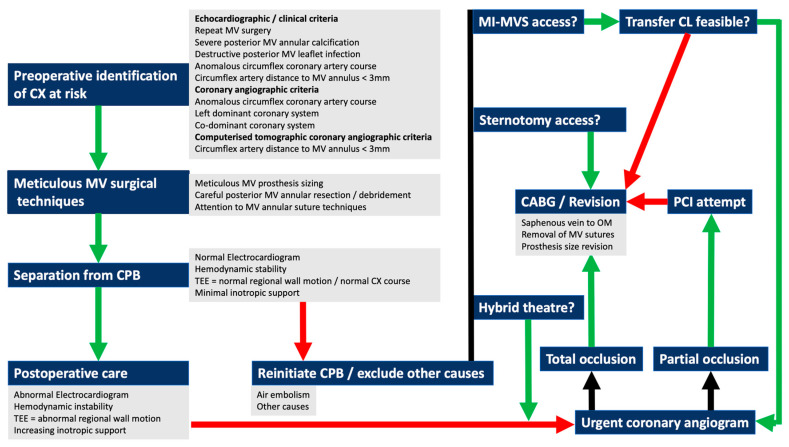
Comprehensive CX injury prevention, diagnosis and treatment algorithm. Abbreviations: CX: circumflex coronary artery; MV: mitral valve; CPB: cardiopulmonary bypass; TEE: transesophageal echocardiography; CL: catheterization laboratory; CABG: coronary artery bypass grafting; PCI: percutaneous coronary intervention. MI-MVS: minimally invasive mitral valve surgery.

The detrimental impact of CX injury during MV surgery on mortality and subsequent quality of life in survivors is well described [1,2,3,4,5,6,7,8,9,10,11,12,13,14,15,16,17,18,19,20,21,22,23,24,25,26,27,28,29,30,31,32,33,34,35,36,37,38,39,40,41,42,43,44,45,46,47,48,49]. Bargagna and colleagues [48] re-emphasized that the true incidence of CX injury is most likely underreported due to unawareness of CX injury in clinically stable patients, publication bias and unrecognized clinical features. Logistical and infrastructure challenges may also contribute to delays in establishing a swift diagnosis. The pathophysiology of acute coronary occlusion and the devastating sequelae of myocardial infarction is extensive studied [88] and reconfirms the importance and urgency in recognizing, diagnosing and treating suspected CX injury during MV surgery as soon as possible. It is imperative that the MV surgeons examine the CX–CS–MV complex in detail as part of their routine valvular heart disease protocols [74,75] with special attention to TTE, TEE, CTA and coronary angiographic-derived images. Anesthetic proficiency in perioperative TEE interpretations is strongly recommended and a thorough CX–CS–MV complex evaluation should be part of the routine. Surgical techniques should follow routine principles with special attention to needle manipulation angles and depth in higher risk zones, meticulous sizing of prosthesis and careful resection and reconstruction of posterior MV components. The operating team should be familiar with the clinical presentation of CX injury and be well prepared to implement a protocol of rapid decision making and treatment. Postoperative continuation of care should also be aware of signs and symptoms suggestive of CX injury and must be able to identify, diagnose and assist with rapid decision making and treatment. A comprehensive CX injury prevention, diagnosis and treatment algorithm is proposed in Figure 6.

## 3. Conclusions

MV interventions are associated with the infrequent risk of iatrogenic CX injury, which requires rapid recognition and appropriate therapy. The preoperative coronary angiography, CTA, TTE and TEE evaluation of coronary artery dominance, CX origin, CX course and the CX–CS–MV complex relationships should be routine. Potential intraoperative maneuvers that increase the risk of CX injury should be avoided where possible and intraoperative suspicions of CX injury should be rapidly confirmed by TEE and treated by either suture release, CABG or PCI. Postoperative CX injury identification must be confirmed by either TEE or coronary angiography with subsequent PCI suggested as being the preferred treatment strategy. Increasing awareness of CX injury during MV surgery is mandatory for the early detection and prompt treatment in an exciting era of increasing minimally invasive surgical and TC interventions.

## Figures and Tables

**Table 1:** Current reports that describe circumflex coronary artery injury during mitral valve surgery.

Author	Year	Procedure	Access	Mechanism	Time of Diagnosis	30-Day Mortality	Treatment Strategy
Danielson [1]	1967	MVR (*n* = 3)	Sterno	Total	PO	Yes	None
Roberts [2]	1969	MVR	Sterno	Total	PO	Yes	None
Morin [3]	1982	MVR	Sterno	Total	PO	Yes	None
Virmani [4]	1982	MVR (*n* = 2) MVr (*n* = 1)	Sterno	Total	PO	Yes	None
Speziale [5]	1998	MVR	Sterno	Total	PO	None	none
Tavilla [6]	1998	MVr	Sterno	Total	IntraO	None	Revision and CABG
Mulpur [7]	2000	MVR	Sterno	Partial	Del	None	MVR
Mantilla [8]	2004	MVr	Sterno	Partial	PO	None	PCI
Sangha [9]	2004	MVr	Sterno	Partial	PO	None	PCI
Nakajima [10]	2005	MVr	Sterno	Total	IntraO	None	CABG
Meursing [11]	2006	MVr	Sterno	Partial	IntraO	None	PCI
Wykrzykowska [12]	2006	MVr	Sterno	Partial	PO	None	PCI
Raza [13]	2006	MVr	MI-MVS	Partial	IntraO	None	PCI
Acar [14]	2007	MVr (*n* = 3)	Sterno	Total (*n* = 3)	IntraO (*n* = 3)	None	CABG (*n* = 3)
Zegdi [15]	2007	MVr	Sterno	Partial	PO	None	CABG
Aubert [16]	2008	MVr	Sterno	Partial	IntraO	None	PCI
Gomes [17]	2008	MVr (*n* = 2)	Sterno	Partial	IntraO	None	Revision
Grande [18]	2008	MVr	MI-MVS	Partial	IntraO	None	PCI
Calafiore [19]	2010	MVr	Sterno	Total	IntraO	None	CABG
Ender [20]	2010	MVr (*n* = 3)	MI-MVS	Partial (*n* = 3)	IntraO (*n* = 3)	None	Revision (*n* = 2) PCI (*n* = 1)
Varela [21]	2011	MVr (*n* = 2)	Sterno	Partial	PO	None	PCI (*n* = 2)
Murugesan [22]	2011	MVr	Sterno	Partial	IntraO	None	Revision
Postorino [23]	2011	MVr	Sterno	Partial	PO	None	PCI
Sheth [24]	2012	MVR	Sterno	Laceration	PO	None	PCI
Schyma [25]	2012	MVR	Sterno	Partial	PO	yes	None
Somekh [26]	2012	MVr	MI-MVS	Total	PO	None	PCI
Ziadi [27]	2014	MVr	Sterno	Partial	Del(5 months)	None	CABG and aneurysmectomy
Folkmann [28]	2014	MVR	MI-MVS	Partial	IntraO	None	PCI
Pettinari [29]	2015	MVr	Sterno	Partial	PO	None	PCI
Monteiro [30]	2016	MVr	Sterno	Partial	Del	None	Revision
Hiltrop [31]	2016	MVr (*n* = 2) MVR (*n* = 2)	MI-MVS (*n* = 2) Sterno (*n* = 2)	Total (*n* = 1) Partial (*n* = 2)	IntraO (*n* = 1) PO (*n* = 2) Del (*n* = 1)	Yes (*n* = 1)	PCI (*n* = 2) CABG (*n* = 2)
Coutinho [32]	2017	MVr (*n* = 6)	Sterno	Partial (*n* = 3) Total (*n* = 3)	PO (*n* = 6)	None	None (*n* = 1) Transplant (*n* = 1) Revision (*n* = 3) PCI (*n* = 1)
Busu [33]	2017	MVr	Sterno	Partial	Del (2 years)	None	PCI
Sunagawa [34]	2017	MVr	Sterno	Total	Del (3 years)	None	PCI
Ahmad [35]	2018	MVr	Sterno	Total	IntraO	Yes	PCI
Gentry [36]	2018	MVR	Sterno	Fistula	Del (1 year)	None	Redo-MVR
Husain [37]	2018	MVR (*n* = 9)	Sterno	Partial (*n* = 3) Total (*n* = 6)	N/A	Yes (*n* = 3)	PCI (*n* = 3) CABG (*n* = 6)
Fortunato [38]	2019	MVr (*n* = 4) MVR (*n* = 1)	Sterno	Total (*n* = 4) Partial (*n* = 1)	PO (*n* = 2) IntraO (*n* =3)	Yes (*n* = 2)	None (*n* = 1) PCI (*n* = 2) CABG (*n* = 1) Revision (*n* = 1)
Scarsini [39]	2020	MVr (*n* = 2)	Sterno	Partial (*n* = 2)	IntraO (*n* = 2)	None	PCI (*n* = 2)
Dello [40]	2020	MVR	Sterno	Partial	IntraO	None	PCI
Felekos [41]	2020	MVr	MI-MVS	Partial	IntraO	None	PCI
Caruso [42]	2020	MVr	MI-MVS	Partial	IntraO	None	Revision
Yavari [43]	2020	MVR	Sterno	Partial	PO	None	PCI (unsuccessful)
Arevalos [44]	2021	MVr	MI-MVS	Total	PO	None	PCI
Gaba [45]	2021	Redo-MVR	Sterno	Total	IntraO	None	PCI
Landa [46]	2021	MVr	MI-MVS	Partial	IntraO	None	PCI
Bulak [47]	2021	MVR	Sterno	Total	PO	None	PCI
Bargagna [48]	2021	MVr (*n* = 5) MVR (*n* = 5)	Sterno	Total (*n* = 4) Partial (*n* = 6)	PO (*n* = 5) IntraO = 5	Yes (*n* = 2)	PCI (*n* = 4) CABG (*n* = 5) Revision (*n* = 1)
Nassereddine [49]	2022	MVr	Sterno	Total	Del (4 weeks)	None	PCI (unsuccessful)

Abbreviations: MVR = Mitral valve replacement; MVr = Mitral valve repair; Sterno = Sternotomy access; MI-MVS = Minimally invasive mitral valve surgery; PO = Postoperative diagnosis; IntraO = Intraoperative diagnosis; Del = Delayed diagnosis; PCI = Percutaneous coronary intervention; CABG = Coronary artery bypass grafting.

**Table 2:** Potential imaging criteria considered to be high risk for CX injury within the context of mitral valve procedures.

Echocardiographic/clinical criteria Repeat mitral valve surgery Severe posterior mitral annular calcification Destructive posterior leaflet infection Anomalous circumflex coronary artery course Circumflex artery distance to mitral valve annulus less than 3 mm Coronary angiographic criteria Anomalous circumflex coronary artery course Left dominant coronary system Codominant coronary system Computerised tomographic coronary angiographic criteria Circumflex artery distance to mitral valve annulus less than 3 mm

**Table 3:** Potential mechanisms of circumflex coronary artery injury during mitral valve surgery.

External compression Oversized prosthesis Hematoma Suture injury Vessel laceration with bleeding Vessel distortion with partial occlusion Vessel occlusion Thermal injury of endothelium Cryoablation injury Radiofrequency ablation injury Embolism Air Bone marrow Fat fragments Suture material Prosthetic material

## Data Availability

The authors are accountable for all aspects of the work in ensuring that questions related to the accuracy or integrity of any part of the work are appropriately investigated and resolved.
